# Creating Clarity and Crew Courage: Preventive and Promotive Measures for a Maritime Industry Without Bullying and Harassment

**DOI:** 10.1007/s41542-022-00129-5

**Published:** 2022-11-03

**Authors:** Magnus Boström, Cecilia Österman

**Affiliations:** Kalmar Maritime Academy, Linnaeus University, SE-391 82, Kalmar, Sweden

**Keywords:** Work environment, Occupational safety and health, Seafarer, Gender equality, World Café

## Abstract

Seafaring shares many characteristics with contemporary working life ashore. However, a major difference is that seafarers can spend up to 12 months aboard a ship that constitutes a work, living and recreational environment. Onboard work includes many stressors that can potentially contribute to workplace bullying and harassment, which in turn can affect safety critical operations. The aim of this study was to identify underlying causes in the organizational and social work environment that can cause workplace bullying and harassment at sea, and to suggest appropriate preventive and promotive strategies and measures. Data were collected mainly through World Café workshops with 56 participants from the Swedish maritime industry. Seafarer occupational health, safety, and wellbeing is largely determined by interdependent factors at micro, meso, and macro levels, where different stakeholders play various roles. Strategies and measures starting at the individual seafarer, and gradually expanding outwards toward the maritime industry are suggested. It is important that a victim of bullying or harassment receives adequate support. Creating crew courage enables employees to both recognize troubling situations and know how to act and respond to a situation. To bridge the gap between policy and practice, the legislative framework needs translating into practical procedures to make sense to the middle manager at the sharp end, with limited knowledge, time, resources, and decision latitude. Future research should evaluate the effectiveness of work environment interventions – what works, for whom, and under which circumstances.

## Introduction

### Working at sea

The international maritime trade continues to grow. In 2020, the global fleet consisted of approximately 99 800 merchant ships (UNCTAD, 2021), crewed by an estimated 1.89 million seafarers (ICS/BIMCO, 2021). The distinctly globalized maritime industry is regulated, financed, operated, and provided with workforce on an international basis. A ship may have its owner in one country, be registered in another, managed and operated in a third, insured in a fourth, and crewed by seafarers from several other countries, often through crewing companies. The challenges in implementing harmonized regulations and labour market conditions are clearly illustrated by the difference between the ships’ ownership, where they are registered, and who works on board. While Japan, Greece and China make up the leading ship-owning economies, Panama, Liberia, and the Marshall Islands are the three largest ship registers, based on both carrying capacity and commercial value (UNCTAD, 2021). At the same time, seafarers are largely recruited from the Philippines, the Russian Federation, and Indonesia with the Philippines being the main supplier of both ratings and officers (ICS/BIMCO, 2021).

Seafarers’ occupational health, safety, and wellbeing share many of the characteristics commonly found in contemporary working life ashore, but there are some unique features. The ship constitutes a work, living as well as recreational environment. Depending on contract, a seafarer can be working on board for as long as a 12-month tour of duty (ILO, 2019, MLC Regulation 2.1). This statutory time limit was further extended when the Covid-19 pandemic made crew changes difficult due to travelling restrictions. During this time, seafarers – often with different national, language, and cultural backgrounds – work, rest, and socialize together.

Although working conditions for seafarers have improved in many respects over the years, working at sea can still be safety-critical, hazardous, and demanding (Oldenburg & Jensen, 2019; Österman et al., 2020; Shan & Neis, 2020). While seafarers’ accident fatalities and injuries have decreased (EMSA, 2021), a scoping review of literature shows an increased concern regarding seafarers mental health status (Jonglertmontree et al., 2022). Lefkowitz and Slade, (2019) report a high prevalence of anxiety and suicidal ideation among seafarers, and a significantly higher prevalence of depression than in other occupational groups, assessed with the same questionnaire. Sampson & Ellis (2020) report that psychiatric disorders are more common on board than before and that mental health problems are higher among seafarers than non-seafarers.

There is not one distinct, conclusive reason that can explain seafarers’ mental ill-health. Some commonly found stressors in the shipboard organizational and social work environment include a sustained workload with limited opportunities for recovery, a continuous need to get to know and adapt to new colleagues and create new working relationships, and not having access to one’s private, social network of family and friends (Allen et al., 2007; Carter, 2005; McVeigh et al., 2019). Other reported stressors among seafarers include perceptions of injustice at industry level, inequities among seafarers, role conflicts, and contradictory demands for cost-effective ship operations (McVeigh, MacLachlan, Coyle, et al., 2019; McVeigh, MacLachlan, Vallières, et al., 2019; Rydstedt & Lundh, 2010).

### Organizational and Social Work Environment

Previous research from various domains shows that these stressors increase the risk of workplace bullying and harassment (Bartlett & Bartlett, 2011; Hoel & Vartia, 2018). Deficiencies in the organizational and social work environment that cause stressful situations, such as high workload, lack of time or insufficient resources to perform the work at hand, have frequently been associated with workplace bullying and harassment (Einarsen et al., 2020; Einarsen & Skogstad, 1996; Leymann, 1996). Perceived injustice may also increase the employees’ vulnerability to bullying (Notelaers et al., 2019), and work-related stress caused by conflicting demands between production and safety can be associated with deviant behaviour (Walsh et al., 2020). As argued by Nielsen (2013), workplace bullying and harassment can create dangerous situations by affecting crew communication, collaboration, and job performance, especially during safety critical operations.

Furthermore, seafaring is a masculine-coded profession with a strong professional culture (Kitada, 2013). Only 2% of all seafarers are women, most of whom work in the service departments in the cruise industry, often in positions associated with lower social status. Women seafarers are more at risk of being subjected to workplace bullying and harassment. As shown by Salin (2021), being a minority of lower social status generally increases that risk. In environments where women challenge the prevailing norms of masculinity, the risk is even more severe (Charles & Grusky, 2005). Another group at heightened risk includes precarious and socially vulnerable workers, since short-term contracts often weaken job security and reduce bargaining powers and other rights (Marín et al., 2021; Rönnblad et al., 2019).

In addition, insignificant leadership, or a complete lack thereof, is a breeding ground for bullying. In work organizations where employees perceive contradictory expectations, or when demands, values and expectations are unclear or unpredictable, there is a heightened risk of bullying, through role-conflict, role-ambiguity, and lack of clear goals at the workplace (Salin & Hoel, 2020). Also, leaders set standards of behaviour by their own actions. Active and constructive leadership decreases the risk of bullying (Salin, 2015). When leaders communicate clear goals and expectations, involve employees in decision-making, show concern for employee needs and employee development, and handle conflicts well, the risk of bullying in the workplace drops significantly. In contrast, laissez-faire leadership, where leaders basically abdicate their leader responsibilities, is associated with higher levels of bullying (Salin & Hoel, 2020). Given the fact that managers are often identified as perpetrators (Österman & Boström, 2022), the impact and importance of leadership is paramount.

### Legislative Study Context

The shipboard work environment is regulated through the International Safety Management (ISM) Code that provides an international standard for the safe management and operation of ships and for pollution prevention (IMO, 2015). The ISM Code requires shipping companies to have a Safety Management System (SMS) in place to ensure that employers take active measures to prevent accidents and ill-health on board. The ISM Code can therefore be seen as setting international minimum requirements.

The study presented herein has been conducted from a Swedish perspective – the data were gathered from Swedish shipping companies and Swedish seafarers. Consequently, in addition to the ISM Code, the work environment is regulated by European directives enacted in national laws. Employment and work on board Swedish flagged vessels is covered by Swedish legislation that is generally stricter than international requirements. In Sweden, the responsibility for the work environment is assigned as a general preventive duty for employers, which in this study corresponds to ship owners. This is established in The Work Environment Act from 1977, a framework act that applies to all areas of occupational life and provides direction in broad terms and sets the goals for achieving a good work environment. A central provision of this piece of legislation is that the work situation and the working environment must be adapted to human needs. The Systematic Work Environment Management provisions (AFS 2001:1) transpose parts of the EC Directive on the introduction of measures to encourage improvements in the safety and health of workers at work (89/391/EEC), for example, concerning the work environment policy, allocation of tasks, expert assistance from outside and written risk assessments.

It is also worth noticing that Sweden has a strong tradition of social dialogue and high level of union representation. The idea of self-regulation through collective bargaining by the social partners is strong. The main agreement from 1938 gives the social partners the right and responsibility to regulate pay and employment conditions. The social partners are often represented in advisory bodies or reference groups to government committees or enquiries. As a result, work environment policies and legislation are consensus oriented. The Work Environment Act emphasizes cooperation in the local systematic work environment management at company level. The Act also specifies that companies with five employees or more should appoint a safety representative. The safety representative is a regular employee who, in addition to their regular work, is tasked to monitor the work environment management at the workplace. To be able to do this, the safety representative is guaranteed adequate training and time, to be included in any action concerning the work environment, and to participate in planning of new premises or changes in existing ones. For this to be realised, cooperation and communication between the employer and safety representatives is paramount.

To complement the traditional focus on physical work environment factors, provisions that explicitly concern the organizational and social work environment (AFS 2015:4) have been implemented in Sweden. For seafarers, these provisions were implemented in 2019. The provisions embrace three main areas: unhealthy workload, working hours, and workplace bullying and harassment. In brief, the provisions stipulate that the employer must organize work and take measures to ensure that demands are balanced with adequate resources and that employees have sufficient time for recovery. Further, work must be planned so that the working hours do not lead to increased risks of accidents and ill-health. Employers also need routines for preventing and managing workplace bullying and harassment. These three areas must be integrated and implemented in company work environment management practices to prevent accidents and ill-health.

### Preventing Workplace Bullying and Harassment

Although the main responsibility of the work environment and prevention of workplace bullying and harassment is placed on the employer, it is possible for individual employees to act in a way to reduce workplace bullying and harassment. The bystander effect, the reduced inclination to help someone in need when in presence of other bystanders, has generally been attributed to three psychological factors: a feeling of reduced responsibility when other people are in attendance; a fear of being judged when helping out; and an idea that if no one else is helping, there cannot really be any acute danger (Hortensius & de Gelder, 2018). Previous literature shows that in-person bystander training, the empowerment of bystanders, has a positive effect on attitudes and behaviours (Mujal et al., 2021). According to Coker et al., (2016), bystander training offers participants skills to recognize potentially violent or troublesome situations, and means to intervene. Thus, bystander interventions can be an effective way for employees to address situations of workplace bullying and harassment, applying a mitigating effect when they occur. A long-term effect of bystander intervention is the development of shared workplace norms and values, and an enhanced responsibility for colleagues’ well-being.

A review of scientific literature on workplace bullying and harassment at sea shows that it remains a substantial problem in the maritime industry (Österman & Boström, 2022). To the extent that workplace bullying and harassment has been considered within the maritime safety management system, it has often been from a reactive stance with emphasis on reporting of incidents. Thus, the responsibility is largely placed on the individual victim, rather than on the organization. While reporting and management of incidents of bullying and harassment inarguably is important, it is not enough. In addition, it is necessary to identify and prevent underlying risk factors in the work environment that constitute a hotbed for workplace bullying, as well as to find and implement promotive measures towards improved work and living conditions for all seafarers.

To add to the knowledge base, this paper presents and discusses findings from four stakeholder workshops focused on the organizational and social work environment on board ships. The workshops were held as part of larger research project, with the overall aim of evaluating existing methods and strategies to reduce the risk of workplace bullying and harassment and strengthen the organizational and social work environment at sea. Specifically, the aim of the workshops reported in this paper was to identify underlying causes in the organizational and social work environment that can cause workplace bullying and harassment at sea, and to suggest appropriate preventive and promotive strategies and measures that can be established by various stakeholders.

## Materials and methods

### The World Café Method

The data presented in this paper were primarily collected using the World Café method, first introduced by Brown & Isaacs (2005) as a way to engage participants into conversations that matter to them, and at the same time provide researchers with rich data. The informal setting, designed to resemble a café, has given the method its name. While the World Café is similar to other methods for collecting qualitative data, such as individual interviews or focus groups, it is an efficient method for collecting ideas and viewpoints from a relatively large group of respondents in a short period of time (Löhr et al., 2020).

The set-up of the World Café sessions followed the outline proposed by Brown & Isaacs (2005) with seven design principles. Table 1 presents these principles together with a brief description of the measures taken in adhering to them during this project. In brief, participants are brought together and after an introduction from the researcher or conversational leader, groups of 4–5 people are assigned to a table. A predetermined topic is discussed, and the group jointly takes notes.

**Table 1:** Design principles of the World Café sessions and their implementation (Brown & Isaacs, 2005)

*General design of a World Café*	*Implementation*
1. Set the context	The participants were informed of the scope of the project and had also received the discussion topics in advance.
2. Create hospitable space	The sessions were conducted in a neutral setting and were preceded by an informal coffee break.
3. Explore questions that matter	Two main themes were introduced before the World Café sessions. A total of eight questions were discussed; however, the nature of the questions allowed participants to steer the discussion in various directions.
4. Encourage everyone’s contribution	Before and during the session, it was stressed that the goal was not to reach consensus, but rather to highlight and discuss an array of diverse ideas. Everyone was encouraged to participate to the best of their ability.
5. Cross-pollinate and connect diverse perspectives	After each discussion, the participants were moved to a new table. The table host briefed the participants about the previous discussion, allowing them to comment, connect, and continue existing ideas.
6. Listen together for patterns, insights, and deeper questions	Having a stationary table host was a means to pick up both similar and repeated thoughts, as well as divergent ideas.
7. Harvest and share collective discoveries	After the small discussions at the café tables, new insights were shared among the participants at a final group discussion. Furthermore, a summarizing report based on the World Café sessions was distributed to the participants.

### Participants and Workshop Procedures

In all, four workshops gathered a total of 56 participants from the Swedish maritime industry (Table 2). The participants were purposefully recruited based on their function and experience within the maritime industry. The category *onboard crew* covers employees working on board a ship, including both officers and non-officers within deck, engine, and service department. Some of the crew members also held a position as safety representative. The category *ship owners* comprises shore-based managers and human resource personnel. A total of 12 ship owners were represented by either onboard or shore-based personnel, together operating a majority of the Swedish merchant fleet. Finally, the category *other shore-based representatives* includes representatives from governmental authorities such as the Swedish Transport Agency, the Coast Guard, the Swedish Maritime Administration, teachers and students from academia, as well as representatives from other industry organizations and maritime trade unions. There was a relatively large proportion of personnel with shore functions as well as safety representatives. Since Sweden has a strong tradition of social dialogue between employer and safety representatives, it was deemed suitable for the study to specifically invite participants that have designated tasks in the systematic work environment work at company level.

**Table 2:** Overview of the number of workshop participants and their main occupation

Workshop	Number of participants (women/men)	Onboard crew	Ship owners	Other shore-based representatives
Workshop 1	7 (4 / 3)	1	1	5
Workshop 2	16 (6 /10)	15	0	1
Workshop 3	16 (8 / 8)	9	2	5
Workshop 4	17 (12 / 5)	5	3	9
**Total**	**56 (30/26)**	**30**	**6**	**20**

Although part of the same project, the four workshops had slightly different foci, which reflected the constellation of the participants. Prior to each workshop, all participants received an invitation with a summary of the topic for the workshop and schedule. Each workshop started with a short introduction to the research project including the background and aim, before presenting the focus of the workshop and what would be expected of the participants, including information about anonymity, confidentiality, and informed consent. The first workshop focused on identifying maritime stakeholders that might impact strategies and decisions regarding preventive and promotive work environment management on board ships. The second workshop addressed the role of safety delegates in onboard preventive work environment management, and consequently the participants were almost exclusively safety delegates working on board ships. The third and fourth workshops explored necessary competences and available tools in the preventive and proactive work.

For workshop 1, an unstructured roundtable setting was used to moderate the participants’ discussion under the guidance of one of the researchers. This was deemed fruitful considering the exploratory task of identifying possible stakeholders. For workshops 2–4, the World Café method was applied. The rooms were furnished for roundtable discussions with tables seating four people facing each other. After an introduction by the researchers outlining the project’s overall purpose and research ethics, two sessions followed with four questions each (Table 3). In the first session, the questions concerned preventive measures, and how the legal responsibilities for the organizational and social work environment can be translated into practice. In the second session, the questions focused on promotive measures, how the work environment work can be elevated from the regulated minimum level.

During these sessions, one person at each table was appointed as “Café host”, with the task of keeping the discussion on track and making sure that everyone got to speak their mind. The hosts were recruited from the participants on a voluntary basis. The host also encouraged everyone to document their thoughts on a joint flipchart with coloured markers. After approximately 10–15 min, the participants moved to other tables, to form a new constellation and discuss a new topic with every move. The host remained at the same table, introducing the topic to a new group. By briefly accounting for what the previous groups had discussed, the dialogue was enriched, and created a shared learning opportunity for all participants. At the end of each session, as well as at the end of the workshop, insights were shared by the whole group in a final summarizing discussion.

**Table 3:** Procedures for workshops 2–4, including allotted time

Step 1–5	Activity	Time
1. Introduction	Presentation of research project and participants, workshop aim, and the World Café Method. Information about what would be expected of the participants, including confidentiality and informed consent.	10 min
2. Session 1 – Identifying and preventing risks in the organizational and social work environment	Discussion of the following four questions: − What information can we use to find out if there are problems with high workload? − If you or a colleague is treated badly at work, how would you like to act and how would you like others to act? What is stopping you? − What skills do managers, safety representatives and employees need to be able to prevent and deal with workplace bullying and harassment? − How do we know if someone is being subjected to workplace bullying and harassment?	45 min
	Summarizing discussion with all participants to share insights and ideas.	15 min
3. Coffee break		15 min
4. Session 2 – Cornerstones in the health-promotive work environment work	Discussion of the following four questions: − How can we work for a more gender equal and inclusive shipping? − What conditions and what support do managers need to be able to take responsibility for the work environment work? − How can we investigate what norms, values and behaviours exist in the workplace? − What can you do to create order in the organization? How can you encourage participation and good communication?	
	Summarizing discussion with all participants to share insights and ideas.	15 min
5. Summarizing discussion and reflections.	Summary of the day, thanking participants.	5 min

### Data Analysis

Workshops 3 and 4 utilized graphic recording to document, enhance, and visualize the discussions. The decision for only using graphic recording for those workshops was based on the focus of the respective workshops. The two first workshops had a narrower focus, while the latter ones identified preventive and promotive measures and thus, were deemed most beneficial of this. Graphic recording is a process to visualize ideas and discussions that stem from group interaction (Dean-Coffey, 2013; Hautopp & Ørngreen, 2018). This can be done in several ways. Here, a live graphic charting was performed by a facilitator in the room. Prior to the workshops, the graphic facilitator was briefed about the set-up and goal of the workshops, as well as the questions that were going to be discussed at the two sessions. A large sheet of paper (about 3 m in length and 1 m in height) was mounted on a wall in the same room as the workshop took place. Based on the initial briefing and the summarizing discussions where all groups shared insights and ideas, the facilitator created a live illustration during the whole duration of the workshop. The purpose of using graphic recording was twofold; it served both as a tool for data analysis, and a tool for communication (Evergreen & Metzner, 2013). As for communication, the graphic recording was a vital element both during the World Café sessions, where it helped the participants visualize their own and others’ ideas, as well as in the dissemination of the results to the maritime industry.

After each workshop, the elicited data, in terms of experiences expressed in words, notes and drawings, as well as the delivery from the graphic facilitator, were systematically summarized and categorized. To create meaning and bring structure to the data, a theoretical thematic approach was adopted (Braun & Clarke, 2006). Two researchers, both with seafaring background as well as practical and theoretical knowledge of systematic work environment management, performed the analysis. Both researchers read the data and categorized them based on the various solutions proposed by the participants and the desired outcomes. Discussions between the researchers further fine-tuned the analysis. Finally, quotes were selected to illustrate the findings. The findings were then grouped into five stakeholder categories to present on which level each measure can be applied. A summarizing report was sent to the participants to provide a learning opportunity and a possibility to apply new ideas within their organizations. In conclusion, the world café method worked as a purposeful learning environment (Ropes et al., 2020), both during and after the workshops.

## Results

The four workshops elicited rich and varied input concerning factors in the work environment that pose a risk for workplace bullying and harassment, and how these may be prevented. It was evident that the participants were able to relate past seafaring and other working experiences to the questions that were discussed. This was manifested as spontaneous storytelling and telling of anecdotes. Tables 4a and 4b present a distilled version of the empirical findings from the World Café workshops. They outline the desired outcome and suggested solutions that were expressed by the participants in response to the questions asked during the workshop (Table 3). The quotes have been chosen to illustrate the data.

The overall theme of session 1 was *risk prevention*. With the theoretical top-down analysis, the coding closely followed the predetermined questions. In other words, this section provides a blueprint of measures to identify and prevent risks in the organizational and social work environment (Table 4a).

**Table 4a:** Session 1 – Identifying and preventing risks in the organizational and social work environment. Compilation of key findings describing desired outcome, proposed solutions, and illustrative quotes from workshop participants

Question	Desired outcome	Proposed solutions	Quotes
What information can we use to find out if there are problems with high workload?	To provide decision support for managers in their continuous work environment work.	Improve structures and means for communication	“Communication is key” “It’s important to have clear expectations” “Pay attention to changed behaviour”
If you or a colleague is treated badly at work, how would you like to act and how would you like others to act? What is stopping you?	To empower employees as bystanders of workplace bullying and harassment.	Bystander training	“Take a stand, now!” “Question unwanted behaviour” “If you do not dare to stand up to bad behaviour, at least try to interrupt the bully” “It’s difficult to know what is ok, and not” “A fear of being the next victim”
	To target a culture of silence. To empower employees to actively participate in the occupational safety and health work, and caring for each other.	Initiatives such as creating *crew courage*, and performing *buddy checks* and *smiley check*s	“Everyone deserves to be a part of the crew” “I’d like my colleagues to support me if I were to be bullied” “As a cadet, you keep silent through fear of not getting a job later on”
What skills do managers, safety representatives and employees need to be able to prevent and deal with workplace bullying and harassment?	To increase awareness of work environment risk factors and consequences of workplace bullying and harassment.	Increased knowledge of: - occupational safety and health in general - workplace bullying and harassment	“Understand your role at, and within, the workplace”
	To help managers meet formal requirements.	Provide time and resources to increase the status of work environment work	“Empower the boss”
	To target the feeling of insecurity and uncertainty, fear of reprisals if intervening or reporting.	Increased knowledge of: - internal routines and procedures for workplace bullying and harassment - where to get support	“A fear of not knowing what will happen”
	To develop a common frame of reference. To create a mutual understanding of safety representative tasks.	Arrange training courses for safety representatives and managers together	“Joint training so that we all ‘speak the same language’”
	To strengthen the safety representatives in their role.	Increased knowledge of: - occupational safety and health legislation - Rights and obligations of safety representatives.	“To be able to plan the safety representative’s work well ahead, these problems need to be prioritised” “Allocation of time!”
	To assist the safety representatives in providing better support to colleagues.	Specific training in: - Conflict management - Handling of difficult situations	“Practical training on how to handle situations of workplace bullying and harassment”
How do we know if someone is being subjected to workplace bullying and harassment?	To facilitate reporting of workplace bullying and harassment.	Clear routines for and increased awareness of reporting workplace bullying and harassment	“If you hear or see something, talk to the bullied person. Don’t wait for it to happen again.” “Trust your gut feeling”
		Whistle-blower system	“There is a need for a robust support structure within the organization”

**Table 4b:** Session 2 – Cornerstones in the health-promotive work environment work. Compilation of key findings describing desired outcome, proposed solutions, and illustrative quotes from workshop participants

Questions	Desired outcome	Proposed solutions	Quotes
How can we work for a more gender equal and inclusive shipping?	To reduce workload, develop and strengthen skills, improve team spirit, counteract gender-marked tasks and lock-in effects.	Ensuring recovery, crewing, working hours, crew composition, increased work rotation through self-governing groups	“Use available technology, for example tools to reduce the need for physical strength”
	To empower women seafarers socially and at work.	Several women on the same ship	“Highlight ships and crews that work actively with inclusion“
	To create a sustainable working life, facilitate for a seagoing career, recruitment and retention.	Improve employment and working conditions for seafarers’ working life	“Make efforts to recruit new groups of people, from other contexts”
	To strengthen fundamental human rights, essential to unlock employees’ full potential.	Develop strategies and implement actions for improved gender equality	“Strengthen norm breaking behaviour”
What conditions and what support do managers need to be able to take responsibility for the work environment work?	To reduce managers’ administrative burden.	Provide usable administrative support systems	“Support is needed from the company’s shore-based functions” “Distribute responsibilities between onboard crew and shore personnel”
	To increase employee cooperation, participation, and influence.	Tools and methods for improved dialogue and reflection of work norms and practices	“Clear support is needed from management, and to make sure that we ‘speak the same language’” “Create interest and promote inspiration. Use a carrot, not a stick!” “The manager provides support, but everyone needs to act”
	To reduce workload and improve task performance.	Being open to new (technical) solutions	“Don’t be afraid to delegate!”
	To create a level playing field for companies and provide support in decision making.	Harmonization and enforcement of legislation	“National authorities, charterers, crewing agencies, they all have demands. Sometimes very different…”
How can we investigate what norms, values and behaviours exist in the workplace?	To create a space where managers, employees and safety representatives interact and discuss norms and acceptable behaviour.	Tools and methods for improved dialogue and reflection of work norms and practices	“Take time to regularly check in with the employees, to listen and watch how they are doing” “Empower the boss”
What can you do to create order in the organization? How can you encourage participation and good communication?	To provide managers with adequate resources and prerequisites for occupational safety and health work in practice.	Clear work descriptions, well defined responsibilities, and mandate to act.	“There is a need for accurate job descriptions, but also that they are followed, revised, and streamlined”
	To reduce managers’ workload.	Review of task allocation and resources.	“There is a need for well-defined job descriptions, and a clear ownership of various tasks”
	To increase trust and job satisfaction and reduce role conflicts.	Clear division of work and responsibilities	“Be the change that you would like to see” “Learn from good examples, and try to focus on positive outcomes”
	To increase performance and create personal wellbeing by reducing uncertainty.	Introduction of new employees when arriving on board.	“Clearly explain why a task has to be done, not just that it must be done”
	To create continuity, establishing and maintaining social relationships.	The same people on the same ship	“Practical experience is important; we learn from each other” “Use the competence that we have on board”

The overall theme of session 2 was *health-promotive work environment work*, aiming to elicit suggested solutions on how to move beyond minimum legal requirements, and towards improved work and living conditions for all seafarers (Table 4b).

Tables 4a and 4b do not indicate who is responsible for implementing and evaluating the effect of the proposed solutions. The reason for this is the general preventive duty assigned to Swedish employers, which makes the employer overall responsible for the work environment. Still, the occupational health, safety, and wellbeing at a personal level is largely determined by interdependent factors at micro, meso and macro levels, where different stakeholders play various roles. Consequently, it is important to look at the stakeholders separately, to examine the work environment from their perspective, and the possible preventive and promotive measures.

### The Employee Perspective

The employee perspective, as presented below, includes the individual employee, but also students during their onboard training at sea. The results from the workshops indicate a heightened feeling of insecurity and uncertainty in the social environment on board. The conversations revolved around the consequences of reporting abusive behaviour, for the victim as well as for an intervening bystander, and there was a fear that the situation would worsen for both. Examples of issues raised by the participants in the dialogues included expected support from colleagues and managers in the wake of an incident, how the individual would be treated afterwards by colleagues, and whether the individual would remain a valued part of the working group.

The participants provided examples of workplaces that were considered to have a prevailing culture of silence, especially regarding workplace bullying and harassment, and equal treatment. This can take the form of, for instance, petty behaviour, gossip, spreading of rumours, and jokes at the expense of others. Other examples were of severe form of workplace violence, including sexual harassment. Situations were described where co-workers experienced an abusive situation but where the victim did not seem to have perceived the situation as abusive. There were also examples of situations where students, during their onboard training, had opted not to raise their voice for fear of not being hired after graduation. This is a particular concern since the Swedish shipping industry is relatively small. Participants further expressed a need for a forum where co-workers can discuss for example attitudes and jargon to create a shared understanding about norms and behaviours in the workplace. Moreover, it is not always clear how and to whom an employee can report an incident or accident of bullying and harassment, nor is everyone aware of who is responsible for handling and investigating such claims and how the investigation will proceed.

*Crew courage, buddy checks* and *smiley checks* were three concepts that were discussed during the workshops as ways to break the prevailing culture of silence and contribute to a favorable organizational culture. These concepts all stress the necessity of actively observing and acting on, for example, behavioral changes in a colleague or malfunctioning interaction within a working group. One proposed measure is the creation of what a participant labelled crew courage. This was discussed in terms of encouraging and enabling actions to support the victim even when others are idly standing by. Buddy checks and smiley checks were suggested as ways to check the mood of each other. Buddy checks are comparable to the safety checks performed by for example scuba divers and divers, and smiley checks are similar to “happiness meters” that are available, for example, after security checks at some airports.

The importance of providing employees with at least some practical and legislative knowledge of work environment was also discussed. A heightened awareness would have several benefits. First, it would provide a better understanding of the consequences of an insufficient organizational and social work environment, and the effects on one’s own health. Second, it would stress in what way individual employees can contribute. In the maritime industry, it has long been common for training initiatives to be carried out digitally through so-called computer-based training (CBT). However, some CBT courses were perceived by participants to be of low quality from a pedagogical standpoint. Further, the problem was raised that it is not always possible to check who is actually completing the training course. It was reported that students who are on board to do their onboard training have been offered compensation for taking compulsory courses for other crew members. In other cases, the correct answers to the knowledge test have been saved in a common folder so that the course can be completed. In cases like these, the result of the training effort is more about “ticking the box” and complying with rules rather than providing increased knowledge.

### The Safety Representative Perspective

Discussions highlighted the necessity of good contact and mutual trust between co-workers and the safety representative. It was stated that when problems related to the work environment arise, an employee might feel more inclined turning to a colleague, safety representative, or union representative, rather than talking directly to a supervisor. In this respect, issues regarding confidentiality were raised. Safety representatives sometimes face the dilemma of promising not to disclose information given in confidence, while still wanting to make the employer aware of problems or shortcomings in the work environment.

A safety representative’s toolbox needs to contain fact-based and declarative knowledge about the scope of workplace bullying and harassment, including relevant legislation, internal policy documents and routines. It is also considered necessary for safety representatives to have a certain amount of social competence, show empathy, and be a good role model.

The cooperation between employers, managers, and safety representatives was perceived as important. There is a perceived added value of having managers and safety representatives attend work environment training together, to develop a common frame of reference and “speak the same language”. Also, there is a need for a mutual understanding of what the safety representative tasks are and how much time that needs to be allocated. To strengthen the safety representatives in their role, requests were further made for in-depth training in conflict management and the opportunity to practice handling of difficult situations.

### The Manager Perspective

Several participants emphasized the importance of managers being given both sufficient knowledge regarding practical work environment management, as well as adequate resources, sufficient time, and tools to be able to handle problems that arise. An organization that seeks to take powerful action regarding organizational and social work environment issues needs to clarify routines and how powers are distributed, so that managers know their areas of responsibility and feel confident in how they should act. Administrative support systems also need to be reviewed. This includes both the usability of the systems themselves, and whether some of the tasks currently performed on board can be taken over by the shore organization to reduce the administrative burden on managers.

Training of managers is a way for the employer to meet formal requirements; still, that does not necessarily ensure that newly acquired knowledge is transformed into practical application. Participants sometimes experience an imbalance between the employer’s goal of providing proper knowledge, and the willingness or opportunity to invest in training with proven effect.

Participation was considered an important part of the promotive work, based on motivation and commitment from both the manager and employees. Clear work descriptions, well defined responsibilities, and mandate to act, have potential to enhance the feeling of ownership of one’s work, thus increasing trust and job satisfaction. Different approaches beyond the traditional “carrot or stick” tactics were discussed to motivate employees. The manager needs to inspire the crew to engage in the work environment work, not simply because they have to, but because they want to.

For managers, coffee breaks were mentioned as a good opportunity to create participation and, in more informal ways, provide and receive feedback about ongoing issues. Through their leadership, managers and supervisors become role models and set norms for the onboard work environment. This requires clarity and that unacceptable behaviours and attitudes receive appropriate consequences.

The participants gave several concrete suggestions for methods and activities that managers can use to engage employees in dialogue about rules and values in the workplace. Some examples were online tools for increased discussion and reflection about common values, and games outlining acceptable and unacceptable jargon and speech. With dialogue exercises and by practicing methods for feedback at work, a learning workplace culture can be developed. A natural and safe approach to feedback on one’s own work provides the possibility of also questioning traditions and routines in order to be able to develop new and more adapted ways of working.

### The Employer Perspective

As a ship is both a workplace and living environment, the boundary between work and private life is blurred. When this extends for longer periods, the feeling of recovery is affected. To some extent, work at sea is also affected by both unpredictable and external factors beyond the control of the crew. Such circumstances might affect the work on board, for example through increased work hours and reduced opportunities for recovery. Long working hours was considered by the participants to affect employees’ participation in social activities on board, which in turn can increase the feeling of exclusion.

A lack of clarity in the structure of work and a vague division of roles and responsibilities can affect the understanding of one’s own part within the workplace. Ambiguous expectations and requirements and an uncertainty about how, and with what resources, the work is to be carried out have consequences. The participants had experienced both cognitive and physical demands in their daily work with potential effects on work performance. Being open to new technical solutions and investigating the possibility of increased work rotation through self-governing groups was highlighted as a mitigating solution. This could develop and strengthen skills, improve team spirit, and counteract gender-marked tasks and lock-in effects. The proposal was deemed of particularly interesting for service personnel on board.

Malfunctioning communication was seen as a contributing factor to deficiencies in the organizational and social work environment and an area that needs to be improved. The messroom is an informal arena where various forms of conversations take place, and sometimes grounds for informal decisions are established, which are later formalized at formal meetings.

It is also important to address the problem of underreporting of workplace bullying and harassment. Clear workplace rules were considered necessary to create space for employees where they dare to speak up, but also to take responsibility for their actions, for example in the event of violations. The employer needs to develop clearer routines for reporting workplace bullying and harassment, and there needs to be an awareness about these routines throughout the workplace. This would also increase the experience of trust towards the employer.

Furthermore, the need for an independent external contact or some form of anonymous whistle-blower system in shipping was discussed to get more people to report incidents and accidents in relation to the organizational and social work environment, especially regarding workplace bullying and harassment.

Three concrete suggestions were raised as to how the employer can work proactively towards a positive work environment on board. Foremost, the employer needs to make sure that all new employees receive proper and thoughtful introduction when arriving on board. This includes work familiarization, socialization, and practical issues such as being provided with working clothes. Secondly, ensuring a certain continuity among the crew so that the same people return to the same ship, is important. Finally, women working in the same shipping company should be placed on the same ship to strengthen them socially and at work.

### The Industry and Societal Perspective

During the workshops, a number of suggestions were put forward regarding what needs to be done from the industry and societal perspective. First, legislative issues were discussed. In a Swedish context, appropriate maritime legislation is in place, even though that area has been slow in conforming to national labour laws. A practical example that was discussed was the regulation regarding organizational and social work environment, including workplace bullying and harassment. This regulation was implemented for Swedish ships three years later than for other shore-based workplaces. As a practical consequence, a ship owner had to follow different rules for their seagoing and shore-based employees, adding to the administrative burden. Managers and employers need to have legislative knowledge. Regulations need to be followed, and violations of the legislation must have consequences. During the workshops, it became evident that reporting of incidents and accidents was considered problematic. There was an uncertainty regarding what should be reported, and to whom and how it should be reported. This leads to underreporting which, in turn, provides authorities with insufficient statistical data, on which inspection and communication efforts are based.

Second, suggestions were put forward on how the infrastructure could be addressed, to improve the working life for seagoing personnel. For example, flexibility in the tour of duty and planning of vacation, the possibility to have time off during holidays, and to bring family members on board were mentioned. Improved support for families, as well as adaptions to the work during pregnancy and the first years of parenthood, are central. Flexibility in day care services and the possibility to temporarily have a shore-based position were two highly valued ideas. Means to communicate with family and friends, such as internet connection, are also important.

Finally, some suggestions dealt with gender equality. There must be working clothes and personal protective equipment that fit all sizes. Managers should also provide opportunities to regularly discuss equality, for example at workplace meetings. Furthermore, ensuring that diverse role models are present throughout the maritime industry is crucial: as lecturers and guest lecturers at schools and universities, and on various positions on board, at both management and operational levels. Similarly, a more diverse view of what it means to be a seafarer, and the skills needed, should also be revised. By doing so, this would provide a larger talent pool for the industry.

## Discussion

As previously noted, the responsibility for the work environment is assigned as a general preventive duty for employers to ensure employees’ safety, health, and wellbeing. In addition to this obligation, the results of this study include several suggested measures that individual employees and managers can take against workplace bullying and harassment. The term crew courage was coined as a maritime version of civil courage (cf. Willems, 2021). Having the courage and the tools to be able to speak up and take action will mitigate the bystander effect (Hortensius & de Gelder, 2018). It is not a personal trait, but rather something that is shaped by clear workplace norms and values, and reinforced by a community of practice (Wenger, 2010). Crew courage provides employees with tools to grab the “bully” by the horns – to deal with a difficult situation directly and confidently. These tools enable employees to both recognize troubling situations and know how to act and respond to a situation. One way for seafarers to gain an increased awareness and measures to address such situations is through bystander training (Coker et al., 2016), and preferably conducted as in-person training (Mujal et al., 2021). This provides a feeling of safety through social support of colleagues and managers. However, to create crew courage, there are numerous prerequisites that need to be in place.

Interrelated factors affecting the onboard work environment can be grouped at micro, meso and macro levels, where the influencing power primarily originates from factors at the macro level. For instance, internationally enforced legislation informs decisions made at lower levels. Since the influential power in the other direction is less pronounced, a sustainable change requires a top-down approach.

The incentives and capacities to act are controlled by the decisions made on the meso level. These include, for example, allocation of resources and prioritizations made by corporate management. Still, the results from this study include several suggested measures that can be taken by onboard managers, employees, and safety representatives at the micro level. However, to prevent bullying and harassment, it is not enough to only focus on what the individual can do. While reporting routines and sporadic training initiatives of employees undoubtedly are necessary, it is not sufficient as the sole solution. Hence, a top-down approach is required. This is in line with previous research, which has shown that maritime authorities (Österman & Boström, 2022) and the shipping business model at large (McVeigh & MacLachlan, 2019) shape the way shipping companies work. In addition, there are three concrete suggestions for how the employer could improve the organizational and social work environment, by addressing issues regarding gender equality, crew continuity, and introduction of new employees.

### Top-down Approach

Even though the employer has the overall responsibility for the working environment, the flag state’s maritime administration has an important task to check compliance with rules. A challenge for both legislative regimes and maritime organizations is the harmonization of international and national legislation to create a level playing field for business. The international regulations state the minimum level for work environment and safety work on board.

In Sweden, where this study is set, there are generally higher requirements. The full introduction of Swedish work environment legislation on Swedish-flagged vessels is considered by the shipping companies to impair competitiveness in the global market. There is potential for the Swedish Transport Agency, in its capacity as the supervisory authority for the working environment, to work internationally for a general increase in the requirements for decent working conditions for all seafarers.

The built-in slowness in the system where the Swedish Transport Agency has to ratify and implement new or amended regulations from the Swedish Work Environment Authority leads to possible complications. It is trying for safety representatives, managers, and employers to know which rules apply to different employees in the same organization, depending on whether they work on board or ashore.

The ambiguity about what applies, what type of incidents are to be reported where, and how, probably contributes to the large underreporting of occupational accidents and ill-health, especially incidents related to the organizational and social work environment. These statistics form the basis for planning of risk-based audits so that they are directed at the greatest work-related risks. The underreporting can negatively affect this planning and prioritization of controls, as well as hinder the organizational learning. In total during the years 2009–2019, only 25 cases of occupational diseases related to the organizational and social work environment were reported within the Swedish shipping industry (Reis & Rydberg, 2020). Thus, there is a need for usable systems and clear reporting procedures for occupational accidents and ill-health, both for formal reports to the administrations and for the employers’ internal investigation.

To increase the image and attractiveness of seafaring, the maritime industry needs to address basic organizational structures and practices that are a breeding ground for workplace bullying and harassment. With the understanding that women seafarers run an extra high risk of being exposed to harassment, all industry actors also need to work long-term for increased gender equality.

### Several women on the same ship

Kanter (1993) argues that in a group with a clear minority that constitutes less than 15% of the group, there is a risk that perceptions and behaviours, such as stereotyping, arise, which create problems for the minority group. This in turn can lead to work environment problems. Having more than one woman in the same team can strengthen the group both socially and at work. Although it takes time to achieve an even gender distribution, one goal needs to be that the women who are recruited do not constitute a clear minority, with the negative consequences that may follow from that (Watts, 2007). There is often a more or less pronounced expectation that women, through their mere presence, can break the prevailing norms and contribute to creating a more heterogeneous work environment. However, the problem is not solved that easily. Women do not want to be treated differently than men. Efforts are required to counteract feelings of both being in the minority and being subordinate (Wahl, 2014).

However, a gender-equal organization is not just about the *number* of women and men that can be solved by a “just add women and stir” strategy (Harding, 1995). It is also about power, influence, status and respect, health, and opportunities provided in the workplace and in the profession. In addition to gender equality, there are other power structures and divisions that include other categories than gender, and these need to be problematized as well. In the organizational processes that create inclusion or exclusion linked to gender, other discriminatory structures are created at the same time. But it is difficult to see the prevailing norms for those who are part of it (Kimmel et al., 2004).

### The same people on the same ship

When it comes to shipping companies’ and staffing companies’ employment agreements, there is potential to improve employment and working conditions for onboard employees. Even if a certain staff turnover is good so that people do not become too accustomed to their behaviors, changing a large part of the crew often results in a heightened burden. A certain continuity among the crew, regardless of nationality, is desirable in order to minimize communication problems that arise as a result of linguistic and cultural differences and to create trustful and sustainable working conditions.

With the same people on board, a mutual learning arises and there is a higher likelihood that common practices become anchored in the organization’s visions, goals, and procedures. This is done through social interactions, but people also learn others’ cultural differences and individual peculiarities, such as gestures and ways of joking. These examples are individual variations that require acclimatization over a period of time. When we know each other, we know about others’ expectations, reactions, and ways of working. An individual’s professional competence depends not only on formal qualifications but also on interaction with those around you. Continuity increases employee loyalty, reduces the risk of having seafarers who are underqualified, and increases employee job security, in addition to the positive aspects it entails for the individual and colleagues in the form of an improved work environment (Bhattacharya, 2015; de Silva et al., 2011; Yuen et al., 2018).

### Organizational Socialization of new Employees

The first time in a new workplace is often a transformative period. In a short time, the new employee must create an understanding of their assignment and role, absorb new theoretical and practical knowledge, and get to know a new work group. The first time is therefore often associated with experiences of uncertainty and stress. To meet this uncertainty and facilitate onboarding, proper introductory efforts are needed that go beyond the mandatory familiarization of ship safety procedures (IMO, 2015). This can, for example, include supervision and sponsorship, and a gradual escalation of tasks. Wanberg (2012) has shown the importance of organizational socialization to facilitate for employees during the first weeks on a new job. A formal presentation and tour have a positive impact on several indicators of a good start in the working life. Career preparation and quality of recovery also prove to be important for a good start. The underlying factor is that the new employee thereby experiences a reduction of uncertainty. Concrete examples of introductory initiatives include receiving feedback on their development, help to reflect on the professional role, help to look at events from different perspectives, and to talk about what it entails to be new.

Well-fitting work clothes and functional protective equipment are also a prerequisite for feeling welcome in a new workplace. Unfortunately, it is not uncommon for such equipment to fit men to a greater extent than women. In addition to the new employee risking feeling insecure, inadequate equipment and poorly fitted work clothes risk negatively affecting their function, thus contributing to safety risks.

### The gap between Policy and Practice

Maritime labor laws clearly state that the responsibility to care for the work environment and provide a workplace free from bullying and harassment, rests on the employer. There is, however, a gap between policy and practice. From a top-down perspective, the broad and over-arching framework legislation places high demand on management since it lacks detailed requirements, thus giving little support for practical work environment management. At the same time, from a bottom-up perspective, a middle manager in the sharp end has limited possibilities to develop and implement a work environment management system, given the limited knowledge, time, resources, or decision latitude.

To bridge the gap between policy and practice – between legislative regimes and middle managers on board – there are several steps that need to be taken. First, employers must deepen their knowledge and understanding of work environment management, and its role for the well-being of employees. Senior managers must also acknowledge that this is something that needs to be addressed, not just to meet legal requirements, but because it benefits everybody. Second, middle managers, in this context the captain and senior officers as the company’s representatives on board, must be provided with the tools they need to work preventively and promotively with the work environment. Finally, for this to be possible, regulations need to be translated into practical procedures. They need to be translated both literally, to more accessible and comprehensible language, and contextually, to make sense to people in their every-day work. This has to be done by individual shipping companies, but preferably in cooperation with other maritime stakeholders, such as trade unions. The gap between policy and practice also serves as an indicator of future research needs. For example, studies that evaluate the effectiveness of work environment interventions, what works, for whom, and under which circumstances (Pawson et al., 1997) are needed. This is important to ensure that the focus of work environment management progresses from sympathetic intentions to measurable impact.

### Study Limitations

When inviting people with various background and experiences to participate in a workshop to share their experiences and opinions, there is always a risk that respondents see it as an opportunity to make their voices heard, hoping that any complaints will be addressed. People who take great interest in their work are more likely to accept the invitation and it is difficult to estimate any potential effects on the attitudinal representativity.

The research design and the choices of methods for data collection and analysis may to some extent be influenced by the researchers’ presuppositions. Having some pre-understanding of, and familiarity with structures, jargon and other peculiarities within the industry can be time saving when planning and performing a research project. Pre-understanding also facilitates acquisition of institutional knowledge of informal hierarchies, cultural values, social interactions and patterns, that otherwise can be difficult to access (Gummesson, 2000). But since the lens through which we view our world inevitably may highlight some, and obscure other components, this pre-understanding can also lead to biased preconceptions (Bryman & Bell, 2007). This aspect has been actively discussed and reflected on by the researchers during all phases of the research work.

The generalizability of the findings obtained in this study might be limited due to its relatively small sample and clear Swedish contextual setting, framed by prevailing national rules and norms that regulate employment and working conditions. However, the study purposedly invited workshop participants with various experiences and responsibilities, representing different views and perspectives. And by adding to the knowledge base, the findings from this study are still relevant also from an international perspective, underlining the need for a systems approach to close the gap between policy and practice.

## Conclusion

When workplace bullying and harassment occurs, it is inarguably important that the victim, and those close by who are immediately affected, receive proper support. Equally important is for managers to have appropriate knowledge and tools to handle the situation. But a lasting change requires efforts that include resources and support at the organizational level of the company, to ensure that bullying and harassment is dealt with systematically. Many factors, however, require a long-term transition of working and employment conditions at industry level. It is crucial that a company that provides a good working environment does not lose its competitive edge, and that all companies within the industry participate on a level playing field.

The results from the World Café workshops show that underlying causes in the organizational and social work environment that can cause workplace bullying and harassment at sea are largely determined by interdependent factors at micro, meso and macro levels. The workshops elicited rich data as well as provided a shared learning opportunity for participants and researchers.

At micro level, from the employee’s perspective, it is central that a victim of bullying and harassment receives support from managers and colleagues. By creating crew courage and strengthen social rapport, a prevailing culture of silence can be targeted. Safety representatives on board have an important role in the work environment work. Their work requires cooperation with employers and managers. By attending training together, particularly when training is conducted as in-person training together with other crew members, a common frame of reference can be developed. The focus should be on quality training and actual knowledge, not just a mean to fulfil legal requirements.

At meso level, through their leadership, managers and supervisors become role models and set norms for the onboard work environment. In order to conduct effective work environment work, managers need routines, powers, but also resources to be able to act clearly. With dialogue exercises and by practicing methods for feedback at work, a learning workplace culture can be developed. The employer has the ultimate responsibility and must ensure that the organizational and social work environment is considered a safety issue. As such, it must be handled within the framework of the safety management system. The employer needs to develop and communicate clear routines for reporting incidents of bullying and harassment.

At macro level, three concrete suggestions were raised regarding gender equality, crew continuity, and introduction of new employees. Having several women on the same ship strengthens the group both socially and at work. Having the same people on the same ship creates continuity among the crew, minimizes communication problems and cultural differences, and creates trustful and sustainable working conditions. Introduction of new employees must go beyond the mandatory familiarization of ship safety procedures and include for example sponsorship and a gradual escalation of tasks.

To move forward from sympathetic intentions to measurable impact, the gap between policy and practice needs to be bridged. This requires a transition in how workplace bullying and harassment is viewed and prioritized, how resources and time are distributed, and that the legislative framework is translated into practical procedures that make sense.

## Data Availability

The data and materials are available upon request.
